# Effect of Experimental Hand Pain on Training-Induced Changes in Motor Performance and Corticospinal Excitability

**DOI:** 10.3390/brainsci7020015

**Published:** 2017-02-04

**Authors:** Nicolas Mavromatis, Cécilia Neige, Martin Gagné, Karen T. Reilly, Catherine Mercier

**Affiliations:** 1 Center for Interdisciplinary Research in Rehabilitation and Social Integration, Québec, QC G1M 2S8, Canada; mavromatis.nico@gmail.com (M.N.); cecilia.neige.1@ulaval.ca (C.N.); martin.gagne.77@gmail.com (M.G.); 2 Department of Rehabilitation, Laval University, Québec, QC G1V 0A6, Canada; 3 ImpAct Team, Lyon Neuroscience Research Center, INSERM U1028, CNRS UMR5292, Bron 69500, France; karen.reilly@inserm.fr; 4 University Claude Bernard Lyon I, Lyon F-69000, France

**Keywords:** transcranial magnetic stimulation, motor cortex, motor acquisition, motor learning, plasticity

## Abstract

Pain influences plasticity within the sensorimotor system and the aim of this study was to assess the effect of pain on changes in motor performance and corticospinal excitability during training for a novel motor task. A total of 30 subjects were allocated to one of two groups (Pain, NoPain) and performed ten training blocks of a visually-guided isometric pinch task. Each block consisted of 15 force sequences, and subjects modulated the force applied to a transducer in order to reach one of five target forces. Pain was induced by applying capsaicin cream to the thumb. Motor performance was assessed by a skill index that measured shifts in the speed–accuracy trade-off function. Neurophysiological measures were taken from the first dorsal interosseous using transcranial magnetic stimulation. Overall, the Pain group performed better throughout the training (*p* = 0.03), but both groups showed similar improvements across training blocks (*p* < 0.001), and there was no significant interaction. Corticospinal excitability in the NoPain group increased halfway through the training, but this was not observed in the Pain group (Time × Group interaction; *p* = 0.01). These results suggest that, even when pain does not negatively impact on the acquisition of a novel motor task, it can affect training-related changes in corticospinal excitability.

## 1. Introduction

Plasticity is the remarkable ability of our nervous system to constantly adapt to changes in our environment or in our body itself, for example, it plays an important role following an injury. From a motor rehabilitation perspective, a better understanding of the neuroplastic mechanisms that underlie motor learning are of particular interest, as it has the potential to translate into strategies to promote the recovery of function. 

Over the last two decades, there has been a large body of research exploring the neuroplastic changes underlying the acquisition of novel motor skills [1,2,3]. However, little attention has been paid to the fact that the plastic potential of a neural network is influenced by its history and/or its current state [4,5]. Some studies in animals have shown that environmental stimuli, such as enriched environments [6,7] or stressful events [8,9,10], affect plasticity. Most human studies have examined state-dependent plasticity using artificial brain stimulation paradigms [4,5]. These studies show that, depending on the conditions, the plastic changes induced by a specific stimulus can be larger than expected, absent, or even opposite to what is expected [4,5]. For example, it has been shown that non-invasive brain stimulation (NIBS) protocols can prime or interfere with the effect of a subsequent NIBS protocol, even in the absence of changes in corticospinal excitability or intracortical inhibition, caused by the priming protocol (see [11,12] for reviews). Other studies have looked at the effect of behavioral activation (e.g., muscle contractions or motor training) prior to plasticity-inducing NIBS protocols (either theta burst or paired-associative stimulation) and have found that it can either suppress, reverse, or increase the modulation of corticospinal excitability observed when the NIBS protocol is employed alone [13,14,15,16]. In order to better understand the behavioral relevance of these state-dependent plasticity phenomena, more studies are needed to assess whether we can expand these findings to contexts in which more “natural” events affect the plastic potential of the human brain, in response to other “natural” events (as opposed to NIBS).

Alterations in sensory inputs have also been shown to affect behavior and plasticity induced by other events. For example, temporary ischemic deafferentation of the hand has been shown to enhance the effect of motor training on a task involving ballistic contraction of the biceps and to increase corticospinal excitability, much more than either motor practice or deafferentation alone [17]. Similarly, Boudreau and colleagues reported that the induction of pain using capsaicin cream significantly interfered with training-induced performance-improvement on a tongue-protrusion task, as well as with the associated increase in corticospinal excitability [18]. Furthermore, the induction of hand pain with capsaicin prior to temporary ischemic deafferentation of the hand increases deafferentation-induced changes in corticospinal excitability [19]. Importantly, in some of these studies the plasticity induced by training or deafferentation is modified even when no significant effect of the alteration in sensory inputs on corticospinal excitability and intracortical inhibition/facilitation is observed [17,19]. Taken together, these results suggest that pain can modulate the plasticity induced by another event.

Pain is present in a large proportion of patients receiving motor rehabilitation interventions, making it a particularly relevant model for studying state-dependent plasticity in relation to motor training. Moreover, nociceptive inputs clearly alter the state of the motor system, leading to a reduction of maximal voluntary contraction, a decrease in endurance, and changes in coordination during dynamic motor tasks, as well as alterations (generally a decrease) in corticospinal excitability measured with transcranial magnetic stimulation (TMS) (see [20,21,22] for reviews). Studies on the effect of local pain on motor learning, however, have produced variable results and have not always replicated the initial study from Boudreau et al. [18], which was completed ten years ago. For example, some report a negative impact on motor acquisition and/or retention [23,24], others show no effect on performance [25,26,27], and some demonstrate positive effects on performance [28,29,30]. Only two of these studies assessed training-related changes in corticospinal excitability [26,27]. Of these two, the results of Rittig-Rasmussen and collaborators [27] appear to be consistent with those of Boudreau et al. [18], as the group trained in the absence of pain had increased excitability after training, while no such increase was observed in the pain group (but no direct comparison between groups was performed). Importantly, despite different patterns of changes in corticospinal excitability, the behavioral improvement in both groups was similar. In contrast, Ingham and colleagues observed no direct effects of local nociceptive input on training-induced plasticity of corticospinal pathways (changes were observed only when pain was applied to a remote location with respect to training) [26]. These authors suggested that the discrepancy between their results and those of Boudreau (who found that pain interfered with training-induced excitability changes) could have occurred if performance during the training task in Boudreau’s study was compromised by subjects using a different strategy to perform the task during pain, i.e., producing less force to avoid pain. However, it should be pointed out that Ingham et al. [26] used a very different task (performing brisk movements of the right index as fast as possible vs. precisely controlling the submaximal force produced with the tongue) and that even in the control group, this task was associated with training-induced changes that were different from those observed in the control group in Boudreau et al. [18]: in Ingham et al. [26] peak-acceleration of TMS-evoked movement was modified during training, but no changes in motor evoked potentials (MEP) were observed, while other studies specifically reported changes in MEPs [18,27]. 

Given the contradictions in the results of previous studies, the objective of the present study was to further investigate whether the presence of nociceptive input applied during training interferes with motor skill acquisition and associated changes in corticospinal excitability. The motor task was selected to attempt to include the advantages of each of the previous studies: (1) a task requiring precise control of force production, as in Boudreau et al. and Rittig-Rasmussen et al. [18,27] (tasks requiring higher precision and control are known to be associated with increased corticospinal control [31]); and (2) a task that permits the total amount of motor practice between groups to be matched, as in Ingham et al. [26]. The capsaicin model was employed as it has previously been shown to produce interference with motor acquisition, retention, and training-induced plasticity [18,23,24]. 

## 2. Materials and Methods

### 2.1. Subjects

A total of 30 healthy subjects were recruited for the main study. Ten additional subjects participated in a preliminary study, the aim of which was to set the parameters for the analysis of the behavioral results (described in Section 2.5.1). All subjects were right-handed and had normal or corrected-to-normal vision, based on a self-report. Subjects were excluded if they presented any history of neurological or psychiatric disorders, pain or musculoskeletal disorders affecting the dominant upper limb, or any contraindications for TMS. The study was approved by the local ethics committee (project #2011-224, approved on 13 May 2011, Institut de réadaptation en déficience physique de Québec, Quebec City QC G1M 2S8, Canada) and subjects provided written informed consent in accordance with the Declaration of Helsinki. After admission to the main study, subjects were randomized to either the Pain (*n* = 15) or NoPain group (*n* = 15).

### 2.2. Experimental Design & Motor Task 

**Figure 1:**
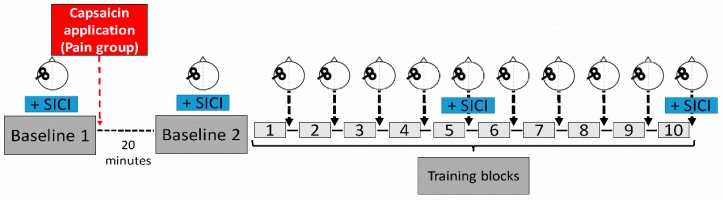
Experimental design. A subject performed ten training blocks of a novel motor task, and each block consisted of 15 successive trials. Corticospinal excitability was assessed using TMS at two baseline measurement periods (prior and after induction of pain (Pain group) or at similar time points (NoPain group), as well as after each training block. Short-interval cortical inhibition (SICI) was measured at the two baseline periods, and after blocks five and ten. The red arrow represents the time of capsaicin application for the Pain group. A wait period of 20 min was imposed on both groups between the two baseline measurements to enable pain levels to significantly increase.

Subjects participated in a single experimental session that lasted approximatively two hours and was comprised of ten training blocks of a novel motor task, as well as a TMS neurophysiological assessment that was performed prior to, during, and after training (see Figure 1). TMS measurements included an assessment of corticospinal excitability (single-pulse TMS) and of short-interval intracortical inhibition (SICI). In order to assess the effect of pain on corticospinal excitability independently of the effect of training, two baseline measurements (Baseline 1 and 2) were performed, one before and one after the induction of pain with capsaicin cream, but both prior to the beginning of training. Corticospinal excitability was then reassessed after each training block, while SICI was reassessed at the halfway point and at the end of training. Throughout the experiment, subjects were comfortably seated in front of a computer screen with their head supported and their right forearm resting on an adjustable armrest, with their elbow flexed at 90°.

**Figure 2:**
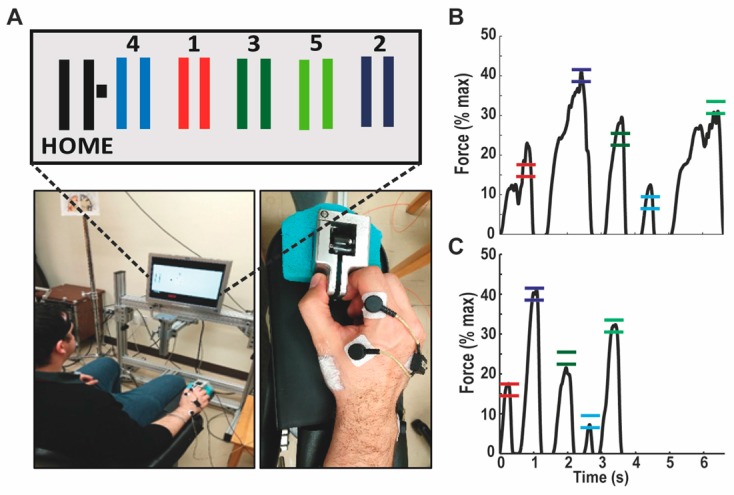
Description of the motor task. (**A**) Pinching a force transducer with the right thumb and index allowed the subject to control the position of the cursor (black square) on the computer screen. The horizontal displacement was normalized against the maximal voluntary force of the subject. Home corresponded to 0% and Gate 2 corresponded to 40% of maximal force. Subjects were required to execute the sequence (Home-1-Home-2-Home-3-Home-4-Home-5-Home) as quickly and as accurately as possible. In addition to the measurement of force, electromyographic activity of the first dorsal interosseous was also recorded. In the Pain group, capsaicin cream was applied over the lateral border of the first metacarpal, as illustrated above (white square proximal to the thumb); (**B**) Example of force trace for a representative trial in the first block of training: accuracy is low, with overshoots for three of the five targets, and the sequence duration is of 6.6 s; (**C**) Example of force trace for a representative trial in the last block of training: the force trace is much smoother, and both the accuracy and speed are improved, with a sequence duration of 3.6 s.

The motor task for which subjects were trained was a modified version of the sequential visual isometric pinch task, developed by Reis et al. [32] (see Figure 2). All subjects were naive to this task and they had to pinch a force transducer between their right thumb and index finger, in order to control the position of a cursor displayed on the computer screen in front of them. They were instructed to move the cursor as quickly and accurately as possible, from the Home position to the five gates that appeared one-by-one on the screen in a set sequence (the sequence was always Home-Gate 1-Home-Gate 2-Home-Gate 3-Home-Gate 4-Home-Gate 5-Home), and the gates were always positioned as depicted in Figure 2A, although they never appeared altogether on the screen. Returning to the Home position triggered the disappearance of the previous gate, as well as the appearance of the next one, even if the subject did not successfully reach the gate during the previous trial. The force needed to reach each gate was normalized according to the maximal voluntary force (MVF) of the subject, which was measured at the beginning of the experiment (average of two trials). From the Home position, the increment in force needed to reach the center of each gate corresponded to 8% of MVF (hence, the center of the rightmost door corresponded to 40% MVF; Figure 2B,C), and the width of each gate corresponded to ±1.6% of MVF. Therefore, the visual gain was matched across subjects in terms of % of MVF, rather than in terms of absolute force. Subjects were given a short familiarization period with the dynamometer, in which they were allowed to reach two static gates that were not part of the trained sequence. Each subject then performed ten training blocks, each of which consisted of 15 sequences (one sequence = five gates). The 15 sequences were performed one after the other in a continuous manner, and subjects were instructed not to stop between each gate or each sequence, as they were required to complete the full block as fast as possible.

### 2.3. Experimental Pain Model

For subjects in the Pain group, cutaneous tonic pain was induced immediately after the first TMS baseline measurement with a single topical application of 1% capsaicin cream. A thin layer (≈2 mm) of cream was applied over the lateral border of the first metacarpal (surface ≈8 cm^2^, see Figure 2), and this was followed by a 20 min waiting period, which has been reported as the time needed to reach a significant increase in pain, as well a significant inhibition of corticospinal excitability [33,34]. Note that the same waiting period was also imposed on the NoPain group. Subjects in both groups were asked to rate their pain on a numerical scale from zero (no pain) to ten (worst pain imaginable) every five minutes during the 20-min waiting period, as well as after the second baseline and after each training block. Topical application of capsaicin cream induces a very stable pain (burning sensation) that is unrelated to movement, and therefore it mimics neuropathic pain more than musculoskeletal pain. As there is no way to alleviate this pain by avoiding a specific movement, it usually does not cause alterations in the execution of well-known motor tasks [23,24], thus allowing the investigation of the effect of pain on mechanisms involved in motor learning, rather than the effect of pain on movement per se.

### 2.4. EMG Recording and Neurophysiological Measures

Electromyographic (EMG) recordings of the right first dorsal interosseous (FDI) muscle were made with surface Ag/AgCl disposable electrodes (1 cm^2^ recording area) in a belly-tendon montage. A ground electrode was placed on the styloid process of the ulna. EMG signals were amplified (×1000), band-pass filtered (20–1000 Hz), and digitized at a sampling rate of 2000 Hz (CED 1401 interface; Cambridge Electronic Design, Cambridge, UK).

Monophasic TMS was applied over the left motor cortex using a 70-mm figure-of-eight coil, connected to two Magstim units through a Bi-stim module (The Magstim Co., Whitland, UK). Positioning of the coil over the head was assisted by a Brainsight neuronavigation system (Brainsight, Rogue Research, Montreal, QC, Canada). Coil orientation was tangential to the scalp with the handle pointing backward and laterally at 45° away from the midsagittal line, resulting in a posterior–anterior-induced current flow, approximately perpendicular to the central sulcus. The hotspot was identified as the site that produced the largest MEPs in the right FDI, at the lowest stimulation intensity. Single pulse TMS intensity was set to evoke an average MEP of approximatively 1 mV with the muscle fully relaxed (mean of 15 MEPs). For the short intracortical inhibition (SICI) assessment, the conditioning stimulus was set at 90% of the active motor threshold (aMT). The active motor threshold was assessed with a contraction of 5% of maximal voluntary contraction, and was defined as the intensity that produced at least 5/10 MEPs with an amplitude greater than 10% of the mean background EMG. The intensity for the SICI test stimulus was similar to the one used for single pulse measurements (e.g., evoking a MEP of 1 mV with the muscle at rest), and this intensity was kept constant throughout the entire experiment [35]. After the second baseline measurement, the subjects began to perform the motor task, and after each training block, 15 single-pulse MEPs were recorded. SICI was also recorded halfway through motor learning (Block 5) and at the end of the motor learning (Block 10).

### 2.5. Data Analysis

#### 2.5.1. Behavioral Variables

Three main variables were used to characterize improvement in behavioral performance: Movement time, Accuracy, and a Skill Measure, designed to account for the speed-accuracy trade-off that is inherent to this type of task. The Movement time variable (reflecting speed as the location of the gates was fixed) consisted of the mean duration of the 15 sequences in a given block (in seconds). As subjects were instructed not to stop in the Home position between successive gates, the total duration of the sequence (from movement onset to returning to the Home position after Gate 5) was measured. The Accuracy variable was defined as the proportion of missed targets within a block. A target was considered as being missed if the cursor went beyond the upper limit of the target (overshoot), or if it returned to the Home position without reaching the lower limit of the target (undershoot). In addition, when a target was missed, the direction of the error (overshoot or undershoot) and the extent by which the target was missed (difference between the maximal % of MVF generated in the trial and the % of MVF needed to reach the closer target limit, positive values representing overshoot), were recorded. The Skill Measure was calculated using the speed-accuracy trade-off equation (see Equation (1)), proposed by Reis et al. [32]. Because our motor task differed slightly from that used in Reis et al.’s study, we evaluated the value of the two independent coefficients (“a” and “b”) from data acquired in a preliminary study of ten subjects (different from those included in the main experiment). Subjects in this preliminary study performed the task at different imposed speeds and in a subsequent experimental session, a subset of these subjects (*n* = 5) completed the whole experimental training protocol, followed by the task with the imposed speeds. From these two sets of data, “a” and “b” were evaluated. Similar to Reis et al.’s observations [32], “a” increased by 81% after training and “b” increased by only 3.7%. This thus confirmed that “a” was suitable to be used as our Skill Measure. The value of “b” used in the equation (b = 3.473) was derived from the mean of “b” from the first (*n* = 10) and second (*n* = 5) data sets.

(1) $$a=\frac{1-error\;rate\;}{error\;rate\;\left(ln{\left(average\;time\right)}^{b}\right)}$$

Equation (1): Estimate of the Skill Measure is represented here by the coefficient “a”. The value of “b” was derived from a preliminary study.

#### 2.5.2. Neurophysiological Variables

In order to quantify changes in corticospinal excitability, the average peak-to-peak amplitude of single-pulse MEPs at each measurement during training was normalized against Baseline 2 (i.e., the measurement taken just prior to the beginning of training—20 min after capsaicin application for the Pain group), using the following equation: (MEP_Training_ − MEP_Baseline 2_)/(MEP_Baseline 2_) × 100. Therefore, the corticospinal excitability measures used for analysis represent a percentage of change from the baseline, with positive values indicating increased excitability and negative values indicating decreased excitability.

SICI was calculated from the average conditioned and test MEP amplitudes, obtained at each time point using the following equation: (MEP_cond_ − MEP_test_)/(MEP_test_) × 100, where negative values reflect inhibition and positive values indicate facilitation. As SICI measures are already expressed as a percentage, no normalization against the baseline was performed.

#### 2.5.3. Statistical Analysis

Student T-tests (two-tailed, unpaired) were performed to compare subject characteristics (age, sex, MVF), as well as the TMS intensity used to evoke a MEP of 1 mV at rest. For most behavioral (Movement time, Accuracy, Skill Measure) and neurophysiological (MEP) dependent variables, three periods of interest were selected, corresponding to three stages of motor training: “Early Training” (first and second training block measures), “Mid Training” (fifth and sixth training block measures), and “Late Training” (ninth and tenth training block measures). A mixed-design two-way ANOVA was then performed on each of these variables, including Group (Pain, NoPain; between-subject factor) and Time (Early, Mid and Late training; within-subject factor) as independent variables. A similar Group × Time ANOVA model was used to analyse the SICI variable, but since fewer time points were available no averaging was performed and a single measurement block was used for each period of interest. It is important to mention that, unlike the other measures, the Early training time point for SICI was measured at Baseline 2, before the start of the training blocks. 

In addition to analysing training-related changes in neurophysiological variables, we also looked for any changes related to the presence of pain. To do this, we performed a mixed-design two-way ANOVA (Group × Time) on corticospinal excitability and SICI data from the two baseline measures.

Exploratory analyses were also performed using Pearson correlations, in order to determine whether changes in behavior and neurophysiological variables over time were associated with initial motor performance (i.e., Skill Measure during Early training).

Finally, to ensure that pain or training did not alter background EMG levels during TMS measurements, the root mean square of the EMG activity in the 100 ms prior to the TMS pulse, was compared between groups and measurement times with a mixed-design two-way ANOVA (Group × Time), using Baseline 1, Baseline 2, Early, Mid, and Late training for the MEP analyses, and Baseline 1, Baseline 2, Mid, and Late training for the SICI analyses.

All statistical analyses were performed using SPSS 22 software (SPSS Inc., Chicago, IL, USA) and post hoc analyses were performed using a Sidak correction for multiple comparisons. Values in parentheses represent the mean ± the standard deviation.

## 3. Results

### 3.1. Group Characteristics

The groups did not differ in terms of age (Pain: 26 years ± 6; NoPain: 27 years ± 6; *t*_(28)_ = 0.498, *p* = 0.622), sex (Pain: 6F/9M; NoPain: 9F/6M; *t*(_28_) = 1.080, *p* = 0.289), or MVF (Pain: 74 N ± 18; NoPain: 71 N ± 19; *t*(_28_) = 0.447, *p* = 0.658). TMS intensity, to elicit a MEP of 1 mV, was not significantly different between groups (*t*(_28_) = 0.159, *p* = 0.875), with an average intensity of 53% (±11) of stimulator output for the NoPain group, compared to 54% (±9.65) for the Pain group.

### 3.2. Pain Rating

**Figure 3:**
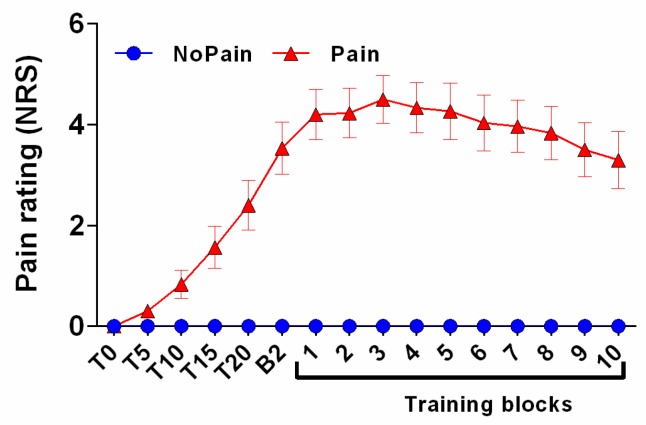
Time course of pain ratings. Subjects rated their pain on a numerical rating scale (NRS) immediately after capsaicin application and then every five minutes. The last measurement before training (B2) was taken right after the second baseline measurement, which corresponded to approximately 25 min after capsaicin application. NoPain group ratings are represented by blue circles and those of the Pain group as red triangles. Error bars represent the standard error of mean (SEM).

Figure 3 illustrates the time course of pain ratings for both groups. In the NoPain group, none of the subjects reported pain at any time throughout the experiment. Pain ratings increased significantly from T0 to Baseline 2 in the Pain group (*t*_(14)_ = 6.798, *p* < 0.001), who had an average pain rating of 3.5/10 just before the beginning of training (corresponding to B2 on Figure 3). Pain ratings then remained stable until the end of the training session (no significant effect of time from B2 to the end of training; *t*_(14)_ = 0.594, *p* = 0.562).

### 3.3. Behavioral Outcomes

**Figure 4:**
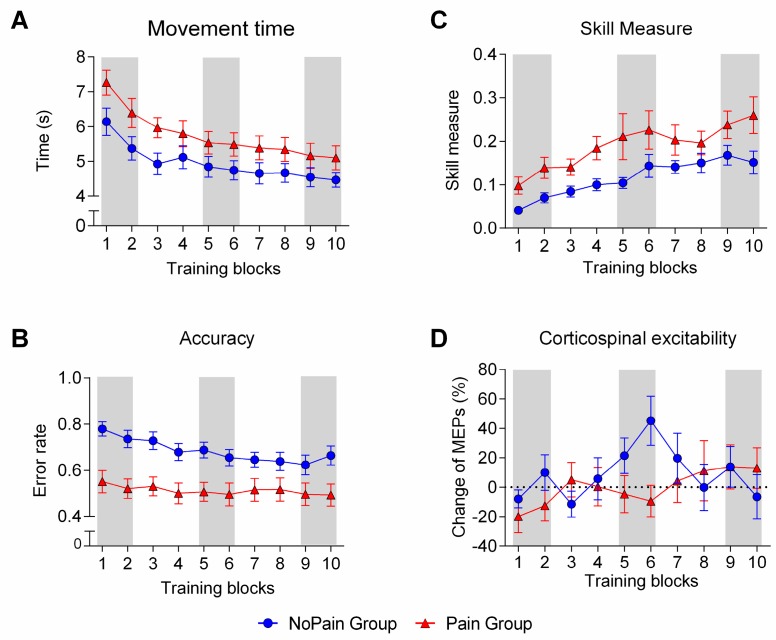
Changes in behavioral and neurophysiological variables over time for each group. (**A**) shows the time course of changes in Movement time (average time needed to perform a sequence); (**B)** presents the time course of changes in Accuracy (average error rate per block of 15 sequences); (**C**) depicts the time course of changes in Skill Measure (an index calculated to take into account the speed-accuracy trade-off); (**D**) illustrates the time course of changes in corticospinal excitability, measured with single pulse TMS. The NoPain group is represented by blue circles and the Pain group by red triangles. Shaded areas indicate the time periods that were used for statistical analysis: Early (training blocks 1 and 2), Mid (training blocks 5 and 6), and Late training (training blocks 9 and 10).

The Movement time analysis (Figure 4A) revealed a main effect of Time (*F*_(2,56)_ = 59.326, *p* < 0.001, *η*^2^ = 0.679), indicating that both groups performed the task faster with training. No significant Time × Group interaction was observed (*F*_(2,56)_ = 1.375, *p* = 0.261, *η*^2^ = 0.047), indicating a similar rate of change in Movement time over time. The Pain group tended to perform the task slightly slower than the NoPain group throughout the training, but this difference did not reach significance (main effect of Group; *F*_(1,28)_ = 3.530, *p* = 0.071, *η*^2^ = 0.11).

The Accuracy analysis (Figure 4B) also showed a main effect of Time (*F*_(2,56)_ = 12.265, *p* < 0.001, *η*^2^ = 0.305) and no significant Time × Group interaction (*F*_(2,56)_ = 2.535, *p* = 0.088, *η*^2^ = 0.083). A main effect of Group (*F*_(1,28)_ = 11.191, *p* = 0.002, *η*^2^ = 0.284) revealed that subjects in the Pain group made significantly fewer errors than those in the NoPain group. Although the number of errors differed between groups, the direction of the errors that were made was comparable, with more than 90% of errors being overshoots (Pain: 90.2%; NoPain: 90.5%). However, the extent by which the target was missed was significantly larger in the NoPain group, no matter whether averaged signed errors (*F*_(1,28)_ = 14.312, *p* = 0.001, *η*^2^ = 0.338) or averaged absolute errors (*F*_(1,28)_ = 8.712, *p* = 0.006, *η*^2^ = 0.024) were compared, reinforcing the idea that the Pain Group was more accurate.

Results for the Skill Measure are presented in Figure 4C. Subjects from both groups showed a significant improvement in performance over time, as revealed by the presence of a main effect of Time (*F*_(2,56)_ = 33.519, *p* < 0.001, *η*^2^ = 0.545). The rate of improvement was similar across groups, as there was no Time × Group interaction (*F*_(2,56)_ = 0.669, *p* = 0.573, *η*^2^ = 0.040). However, a significant main effect of Group indicated that subjects in the Pain group performed better throughout the training period (*F*_(1,28)_ = 5.268, *p* = 0.029, *η*^2^ = 0.158). This finding is consistent with the superior Accuracy of the Pain group (i.e., lower error rate), in the absence of a significant difference between groups in Movement time.

### 3.4. Neurophysiological Outcomes

#### 3.4.1. Pain-Related Changes

The analysis of corticospinal excitability at Baseline 1 and 2 revealed no significant effect of Time (*F*_(1,28)_ = 0.098, *p* = 0.757, *η*^2^ = 0.003), Group (*F*_(1,28)_ = 0.173, *p* = 0.681, *η*^2^ = 0.006), or Time × Group interaction (*F*_(1,28)_ = 3.047, *p* = 0.092, *η*^2^ = 0.098). This indicates that the application of capsaicin per se had no effect on corticospinal excitability, and that both groups had comparable MEP amplitudes prior to the beginning of training (Pain group: Baseline 1 = 1.07 mV ± 0.37, Baseline 2 = 1.26 mV ± 0.81; NoPain group: Baseline 1 = 1.31 mV ± 0.79, Baseline 2 = 1.17 mV ± 0.6). 

Analyses of SICI, comparing Baseline 1 and 2, revealed no significant effect of Time (*F*_(1,26)_ = 0.086, *p* = 0.771, *η*^2^ = 0.003), Group (*F*_(1,26)_ = 0.457, *p* = 0.505, *η*^2^ = 0.017), or a Time × Group interaction (*F*_(1,26)_ = 0.027, *p* = 0.872, *η*^2^ = 0.001), indicating that the presence of pain had no effect on SICI and that both groups had comparable SICI levels prior to the beginning of training. (Pain group: Baseline 1 = 65.9% ± 32.8%, Baseline 2 = 67.5% ± 31.5%; NoPain group: Baseline 1 = 72.7% ± 20.25%, Baseline 2 = 73.15% ± 15.22%). 

#### 3.4.2. Training-Related Changes 

The analysis of change in corticospinal excitability during training (Figure 4D) revealed a significant Time × Group interaction (*F*_(2,56)_ = 4.815, *p* = 0.012, *η*^2^ = 0.147). Post-hoc analyses revealed that changes in corticospinal excitability were only significantly different between groups at Mid training (*p* = 0.019). Looking at changes over time within each group, the NoPain group showed a significant difference between the Early and Mid training periods (*p* = 0.005), followed by a return to the baseline in the Late training period (Early vs. Late: *p* = 0.995). This increase in MEPs, from Early to Mid training, was not observed in the Pain group (*p* = 0.713), which showed no significant differences between any time periods. Nevertheless, MEPs in the Pain group slowly increased across time and tended to be greater at Late training compared to Early training (*p* = 0.072), which may indicate a more gradual rise in corticospinal excitability than in the NoPain group.

Analysis of SICI over time during motor training revealed no significant effect of Group (*F*_(2,27)_ = 1.031, *p* = 0.319, *η*^2^ = 0.037), Time (*F*_(2,54)_ = 0.334, *p* = 0.717, *η*^2^ = 0.012), or a Time × Group interaction (*F*_(2,54)_ = 0.017, *p* = 0.983, *η*^2^ = 0.001). 

### 3.5. Correlational Analyses between Initial Performance and Training-Related Changes 

**Figure 5:**
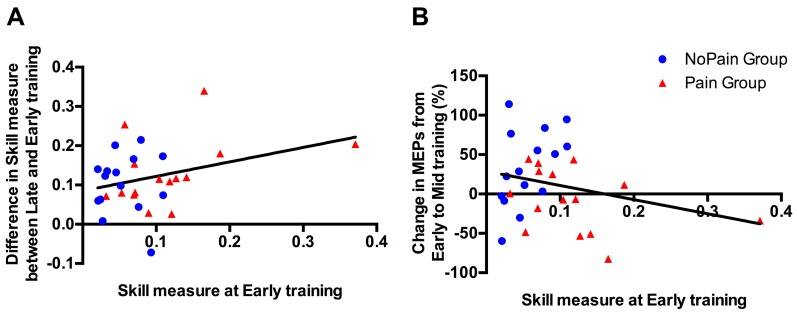
Association between Skill Measure during Early training and (**A**) improvement in Skill Measure from Early to Late training; (**B**) changes in motor evoked potential (MEP) amplitude (%) at Mid training relative to Early training. Blue circles represent subjects from the NoPain group and red triangles subjects from the Pain group. The regression lines have been fitted on data from both groups.

As a significant difference was observed between groups in the Early training phase, correlational analyses were performed to explore whether this difference in initial performance in Skill Measure, was likely to impact on subsequent behavioral improvement (i.e., difference in Skill Measure between Late and Early training performance), or on changes in MEP amplitude (i.e., % of change between Mid (where changes were observed in the Control Group) and Early training). As depicted in Figure 5, no significant correlation was found for either of the behavioral changes (*p* = 0.090; *r* = 0.315), or the corticospinal excitability changes (*p* = 0.173; *r* = −0.255).

### 3.6. Background EMG Levels throughout the Experiment

Analyses performed on pre-TMS pulse background EMG levels revealed no effect of Group, Time, or Time × Group interaction (all *p* values >0.13), indicating that the changes in MEP amplitude reported above cannot be attributed to changes in background EMG related to the motor activation required to perform the task.

## 4. Discussion

More than ten years ago, Boudreau and colleagues proposed that the presence of acute (experimental) pain during training of a novel motor task interferes with motor skill acquisition and associated changes in excitability [18]. This idea is controversial, however, as subsequent studies have produced contradictory results, at both behavioral and neurophysiological levels [26,27]. The results of the present study suggest that pain does not interfere with the acquisition of a new motor task, but that it does eliminate, or at least slow-down, training-related changes in corticospinal excitability.

Interestingly, even though pain altered training-induced corticospinal excitability changes, our behavioral results suggest that the Pain group’s performance was superior to that of the NoPain group. Indeed, throughout training, the Skill Measure was higher in the Pain group, as the two groups had similar Movement times, but the Pain group was more Accurate (fewer errors and smaller error sizes). Importantly, the Pain group outperformed the NoPain group right from the beginning of training, rather than showing a faster rate of improvement (i.e., no significant Time × Group interaction was observed, meaning that there was no difference in acquisition once the initial difference in performance was accounted for using intra-subject comparisons). This suggests that Pain did not impact on the motor acquisition process itself, but rather potentially affected performance through more cognitive processes such as strategy selection or attention to the task. 

Although superior performance in the presence of pain appears counter-intuitive, the same pattern of results has also been reported in two recent studies looking at the effect of acute pain induced with capsaicin on a sequence typing task [29] or a tracing task [30]. The results of the first study were similar to ours in that they showed a difference in accuracy between groups at the first exposure to the task (with better accuracy in the pain group, and no difference in speed) [29]. In contrast with our study, however, they observed no differences between groups at the end of the training, potentially because of a ceiling effect related to the task selected. The results of the second study, using a more challenging task, also revealed superior accuracy in the pain group at the beginning of training, but that difference was still present at the end of training [30]. Furthermore, they observed no differences between groups for the percentage of change in motor errors during acquisition—a pattern extremely similar to our results.

Contrary to what has been previously reported [33,34], we observed no direct effect of pain on neurophysiological variables measured before training began. First, there was no inhibition of MEPs 20 to 25 min after the application of capsaicin. This is surprising given that the modality, location, and intensity of pain used in our study and in previous studies was very similar, and since the stimulation intensity and targeted muscle were either the same or physically close (i.e., FDI vs. Abductor Pollicis Brevis muscle). It should be pointed out that both of the previous studies showing MEP inhibition were performed on small sample sizes (*n* = 7 and 11), without the use of a neuronavigation system and without a pain-free control group. Note, however, that even if we make a direct comparison of MEPs prior to and 20 min after capsaicin application in only the Pain group, we do not observe a significant change in MEP amplitude (*t*_(14)_ = 1.286, *p* = 0.219). Second, the presence of Pain did not affect SICI. Again, this result contrasts with the only previous study looking at SICI using the capsaicin model [34] (albeit with a small sample size), but is consistent with several other studies with larger sample sizes that observed no changes in SICI using other acute pain models (joint or muscle pain) [36,37,38]. 

Although pain did not affect neurophysiological measures obtained before training, modulations in corticospinal excitability were observed throughout training. In the NoPain group, corticospinal excitability reached a peak midway through the training, and then came back to the baseline values. Such transient increases in corticospinal excitability during the early stage of learning followed by a decrease in MEP amplitude (despite the maintenance—or even further improvement—of performance) have previously been reported by studies using motor tasks, such as a serial reaction time test [39] or a ballistic pinch task [40]. The reason for observing this specific timing of a transient increase in MEP amplitude (and subsequent decrease) is not fully clear. At the moment at which the increase was observed (Mid training), subjects in the NoPain group had already reached 73.5% of their total motor gains (i.e., change in Skill Measure between Early and Mid training, relative to the change from Early to Late training). The peak of corticospinal excitability (or cortical map) changes has been proposed to be associated to the transition from implicit to explicit knowledge of a motor sequence, or more generally, because of a change in motor strategy (less reactive and more anticipatory) [39].

Even if the changes in the NoPain group are consistent with previous observations in the literature, it is difficult to explain why these learning-related changes in MEPs are not seen in the Pain group, or occur more slowly. This difference between groups with respect to MEP changes is particularly surprising in light of the fact that the rate of improvement was similar across groups for all behavioral variables (as revealed by the absence of a Time × Group interaction), despite the superior performance of the Pain group from the very beginning and throughout training. One could argue that the lack of MEP changes could be explained by superior performance from the very beginning in the Pain group, leaving less potential for improvement. This appears unlikely, however, as: (1) no ceiling effect was observed in performance for the Pain Group (i.e., the amount of improvement was similar to that of the NoPain Group, and a higher initial performance was not predictive of less improvement over time); (2) subjects were compared to themselves over time using intra-subject analyses; and (3) correlational analyses showed no relationship between the amount of change in MEPs from Early to Mid Training and the Skill Measure during Early training. The difference in performance in the Early Training phase remains a limitation, however, and a replication of the results showing differences in corticospinal excitability across groups is warranted, in order to ensure the validity of this finding. 

The observations described above, that initial training-related changes in cortical plasticity dissipate even though performance is maintained [39,40], illustrate the fact that changes in behavior and corticospinal plasticity are not necessarily always closely associated. This is consistent with a study on force-field adaptation, showing that changes in corticospinal excitability while learning to compensate for a perturbation are observed when the exposure to the force-field is introduced abruptly but not when it is introduced gradually over many trials, despite the fact that the final performance is similar [41]. The task used in the present study was quite different from force-field adaptation, so no direct comparison can be made. Nevertheless, the force-field example shows that applying different learning strategies might lead to similar behavioral improvement while relying on different neural processes. Another study showed that ballistic but not ramp practice was associated with increased TMS-evoked MEP amplitudes, despite the fact that the feedback and effort was similar between both tasks [40]. Although both groups received similar instructions in our study, the trend for a difference in Movement time suggests that the Pain group favored accuracy over speed, leading to less ballistic force production. It is important to keep this in mind when comparing the results to those of other studies in which adoption of an alternative speed-accuracy strategy is not possible, for example when the timing of force production is dictated by the task rather than self-selected (such as in Boudreau et al.’s study [18]). 

The spontaneous use of a different motor strategy in subjects training in the presence of pain has recently been reported in force-field adaptation studies involving either reaching [24] or gait [42], and does not necessarily impact on global performance [42]. The possibility that the presence of pain leads subjects to use a different strategy or to rely on different sensorimotor processes during learning, is also supported by recent observations showing differences in early cortical somatosensory-evoked potentials, following motor learning acquisition in subjects trained in the presence of pain (induced with capsaicin) vs. pain-free subjects [28,29,30]. If pain impacts more on strategy selection than on the learning capacity per se, it appears likely that its effect would be easier to detect in more complex tasks, that involve a larger number of degrees of freedom (most studies, like the present study, use tasks with a relatively low level of complexity in order to allow the measurement of changes in corticospinal excitability in a specific muscle [18,26,27]).

An alternative explanation for our results could be that the presence of pain increased attention to the trained hand or to the task, and that attention, rather than pain, explains the difference between groups in terms of performance and corticospinal excitability modulation throughout training. While this hypothesis could explain the behavioural results (Pain group performing better because they pay more attention), it is difficult to reconcile with our observation of less facilitation of corticospinal excitability. Indeed, previous findings have reported greater facilitation in response to paired associative stimulation paradigms when attention was explicitly directed toward the tested hand [43,44]. However, it is also possible that increased attention to the painful hand impacts on the motor strategy selection (for instance favoring accuracy over speed) rather than acting directly on corticospinal excitability.

## 5. Conclusions

In conclusion, the results of the present study show that pain can affect training-related changes in corticospinal excitability. This can occur without interfering with performance improvement during motor skill acquisition, as our Pain group displayed a superior performance from the very beginning of the training and had a rate of improvement over time which was similar to the NoPain group. Overall, these results suggest that subjects trained in the presence of pain employ different strategies or neural processes to support motor skill acquisition. It remains to be confirmed whether these alternative strategies/neural processes have carry-over effects in terms of retention or transfer, as only a few studies have investigated these questions, with variable patterns of results [23,24,29,30,42].
